# Environmental Enrichment Enhances Cerebellar Compensation and Develops Cerebellar Reserve

**DOI:** 10.3390/ijerph19095697

**Published:** 2022-05-07

**Authors:** Francesca Gelfo, Laura Petrosini

**Affiliations:** 1 Department of Human Sciences, Guglielmo Marconi University, Via Plinio 44, 00193 Rome, Italy; 2 IRCCS Fondazione Santa Lucia, Via Ardeatina 306, 00179 Rome, Italy; laura.petrosini@uniroma1.it

**Keywords:** environmental enrichment, cerebellum, cerebellar reserve, cognition, neuroplasticity, neuroprotection, compensation, recovery of functions, animal models, rodents

## Abstract

The brain is able to change its structure and function in response to environmental stimulations. Several human and animal studies have documented that enhanced stimulations provide individuals with strengthened brain structure and function that allow them to better cope with damage. In this framework, studies based on the exposure of animals to environmental enrichment (EE) have provided indications of the mechanisms involved in such a beneficial action. The cerebellum is a very plastic brain region that responds to every experience with deep structural and functional rearrangement. The present review specifically aims to collect and synthesize the evidence provided by animal models on EE exposure effects on cerebellar structure and function by considering the studies on healthy subjects and on animals exposed to EE both before and after damage involving cerebellar functionality. On the whole, the evidence supports the role of EE in enhancing cerebellar compensation and developing cerebellar reserve. However, since studies addressing this issue are still scarce, large areas of inconsistency and lack of clarity remain. Further studies are required to provide suggestions on possible mechanisms of enhancement of compensatory responses in human patients following cerebellar damage.

## 1. Introduction

The brain is characterized by the now-recognized capacity of changing its own structure and function in response to stimulations that can come from both internal and external environments, which is known as *neuroplasticity* [1,2]. Structural and functional plastic rearrangements are triggered by the transmission of information through neuronal circuitries, which react to each experience by circulating electrochemical signals. Such a plastic reorganization occurs during neural system development, when the brain is engaged in learning and memory, and also when the nervous system undergoes damage [3]. In fact, the brain always tends to respond with the maximum possible compensation for damage by reorganizing itself in order to recover as much function as possible and to reach a new homeostatic equilibrium [4,5].

In the context of the studies focused on neuroplasticity, the *reserve hypothesis* has been formulated. This theory affirms that the experiences that individuals encounter throughout their life affect and fine-tune brain structure and function, providing the individuals with a resilient apparatus [6]. As first observation, it was described that patients affected by Alzheimer’s disease with different life experiences exhibited symptoms in association with different levels of neural injury or degeneration, thus showing different susceptibility to brain damage [7]. This concept has then been enlarged to a wide range of pathological conditions [8]. The concept of *reserve* evolved over the years; originally articulated in two components—the brain reserve and the cognitive reserve—it is now described as a multifactorial frame. The *brain reserve* conceptualizes the evidence that an individual that is provided with a more and better structured brain is able to more resiliently face damage. This principle applies to all the components of cerebral structure at molecular and supramolecular levels, such as brain weight and volume, neuron number, neuronal morphology, density and morphology of neuroglia and synapses, structure of circulatory system, neurotransmitters, and neurotrophic factors [9,10,11]. On the other hand, the *cognitive reserve* is tightly linked to cognitive processes. It can be described as a high-level capacity to efficiently engage the residual functions in order to fulfill tasks and address daily activities [9,10,11]. The *neural reserve* is intended as a kind of summa of the two previous concepts and regards the ability to efficiently engage neuronal networks and alternative strategies in cognitive performance [9,10,11]. Recently, the concept of *brain maintenance* has been included in this framework. This idea is somehow similar to the one of brain reserve, and accounts for the capacity of genetics and lifestyle to protect the brain from the development and accumulation of pathological changes [9,10,11]. Finally, it is noteworthy that all these concepts are also linked to the capacity of the brain of *compensation*, which is the ability of the brain to cope with damage by reacquiring as much function as possible [12].

A fundamental issue on which the studies in this field have focused regards the investigation of the experiential factors that are able to act as “reserve-builders”. Fundamentally, three dimensions are understood to be mainly involved: the cognitive factor, the social factor, and the physical factor [9,10,13]. The *cognitive factor* regards all the activities that involve the individual by requiring a high-level mental investment. Basically, educational level and job complexity are considered, but a large range of leisure activities may be included in this aspect [14,15,16,17]. The *social factor* regards the social networks in which the individual is involved. All social relationships fall in this category, in a large range that includes familiar status, parentage, friendship, etc. [18,19]. The *physical factor* regards the habits that constitute the lifestyle of the individual, such as physical activity, diet, smoking, sleep, alcohol intake, and consumption of beneficial dietary elements [20,21,22].

In animal models, the effects of the enhancement of these three factors on brain structure and function has been studied by using the environmental enrichment (EE) experimental paradigm. The EE paradigm was introduced in the sixties and then widely used with rodents by comparing animals reared in an enriched environment with animals reared in standard laboratory housing conditions [23,24]. By means of this paradigm, it is possible to manipulate the variables concerning rearing conditions with a high-level control. In this way, it is possible to construct a specific design in which to test the effects of a single factor or a combination of factors; to determine the starting, the duration, and the end of the exposure period; to choose the way in which one manipulates each factor (e.g., by stimulating only a sensory channel or more than one in association); and to exactly establish the characteristics of the animals exposed to EE (e.g., species, age, gender, healthy or pathological conditions, etc.) [9,25,26]. Basically, in animals the *cognitive factor* is mimicked by making the rearing environment more complex, providing the cage with a number of objects of various natures, shapes, sizes, and colors, which are frequently rearranged and replaced, in order to enhance the exposure to novelty; the *social factor* is mimicked by modulating the number of individuals that are reared in the same cage, typically augmenting the quantity in comparison to the minimum for laboratory standard; the *physical factor* is mimicked by using cages bigger than the standard ones and equipped with shelves, ladders, and running wheels (frequently indicated as a key-element), to stimulate motor activity and explorative behavior. In addition, specific supplementary diets may be administered [13,26,27,28].

Several studies have used the EE paradigm to model the lifespan experiences of individuals. Consequently, structural and functional cerebral effects of EE have been studied in healthy animals and also in pathological models. In this way, the *neuroprotective* effects of highly stimulating life experiences have been studied by exposing animals early to a more or less lengthy period of EE *before* the occurrence of brain damage. Furthermore, the EE paradigm has been used as a versatile paradigm of *therapeutic* non-pharmacological treatments (useful to enhance spontaneous brain compensation abilities) by exposing animals to a more or less lengthy period of EE *after* the occurrence of brain damage. On the whole, substantial evidence has been provided by the studies based on the EE paradigm about the experience-empowering impact of EE on the entire brain structure, at both molecular and supramolecular levels [27,29,30,31,32,33,34]. Moreover, motor, behavioral, and cognitive functions have been reported to be improved by the exposure to an enriched environment [26,35,36,37,38]. However, several issues remain open regarding the systematic effects of EE on specific processes and brain regions in relation to the healthy or pathological condition of animals [10,27,29].

A brain region known to greatly respond to somatosensory integration and control with plastic rearrangement, including in adult age, is the cerebellum. This cerebral area is classically known to support high-level cognitive and emotional abilities (such as learning and memory processes, spatial cognition, language, reasoning, emotions, and mood) by recursively rearranging its complex connections with the cortical and sub-cortical regions [39,40,41,42,43,44,45,46,47,48,49,50,51]. Moreover, several studies have documented the cerebellar capacity to compensate deficits derived from damages of multifarious nature [52,53,54,55,56]. Numerous mechanisms at both cellular and sub-cellular levels are involved in such a plastic re-adaptation [4,52,55,57,58,59]. Consequently, it is of great interest to analyze how the environment interacts with the predisposition of the cerebellum to recover functions in the presence of damage. The EE paradigm appears to be an ideal tool for such investigations. Interestingly, Cutuli et al. [60] evaluated the effects of two different EE protocols by exposing animals to an enriched environment only before or only after the ablation of a half of the cerebellum (hemicerebellectomy). By investigating postural and locomotor behaviors, in association with striatal synaptic activity and morphology of interneurons, the authors documented that the exposure to EE exerted beneficial effects on the compensation of the cerebellar deficits when the exposure to an enriched environment occurred both before and after the damage.

In this framework, the present review specifically aims to collect and synthesize the evidence provided by animal models on the EE effects on cerebellar structure and function by taking into account the studies on healthy subjects and on animals exposed to EE both before and after damage involving cerebellar functionality.

## 2. Methodology of Literature Search

A methodical literature search was conducted in PubMed and Embase databases by screening all titles and abstracts obtained by searching for the combination of the “environmental enrichment” OR “enriched environment” AND “cerebell*” key-words. Moreover, full texts and reference lists were screened to identify further potentially relevant articles. Articles fulfilling the following criteria were included in the present overview:

i. as *population of interest*, we selected rodents, both healthy subjects and pathological models;
ii. as *intervention of interest*, we selected the exposure to multidimensional EE in a period between birth and death (in healthy conditions, before damage, or after damage). As for pathological models based on genetic manipulations, the exposure to EE was considered as preceding the damage when it occurred in early life, before the onset of disease symptoms; it was considered as following the damage when it occurred later in life, after the onset of disease symptoms;
iii. as *control group of interest*, we selected animals reared in standard laboratory conditions;
iv. as *outcomes of interest*, we selected structural and functional cerebellar effects of rearing conditions. It is worth emphasizing that only studies analyzing at least one (structural, physiological, or biological) cerebellar effect of rearing conditions were included. Cognitive and behavioral effects were considered of interest only when associated with a cerebellar correlate.

No language limitation was selected. No publication period limitation was selected. Records indexed up to February, 2022 were screened.

**Figure 1:**
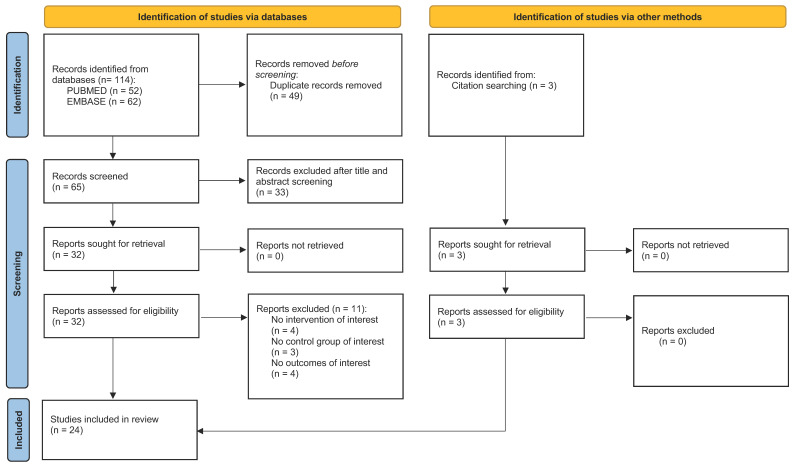
PRISMA flow-diagram [61] illustrating the literature search process.

We identified 114 records from databases. After duplicate record removing, we screened titles and abstracts of 65 papers. After title and abstract screening, we assessed 32 studies for eligibility. Among these, 11 were excluded, since they did not meet our inclusion criteria. Moreover, 3 eligible studies were obtained from citation searching. Consequently, 24 relevant papers (12 on healthy subjects; 5 on subjects exposed to EE before the damage; 7 on subjects exposed to EE after the damage) that met the inclusion criteria were considered for the present review. Figure 1 shows a detailed flow-diagram of the literature search conducted in accordance to the Preferred Reporting Items for Systematic Reviews and Meta-Analyses (PRISMA) recommendations [61].

We collected the following data: authors; year of publication; animal species; pathological model, when present; animals’ age or weight at the start of the exposure to EE; EE type (by specifically noting if the paradigm encompasses running wheels and novelty manipulation); EE duration; EE effects on cerebellar structure and function; animal age at the effects evaluation. As for cerebellar correlates, when not specified, the evaluation regarded the entire region.

All data collected are illustrated in Tables.

## 3. Environmental Enrichment Effects in the Cerebellum of Healthy Animals

Most studies dedicated to cerebellar effects of EE in healthy animals were focused on *early* exposure, that is, when the rearing of the subjects in an enriched environment started from weaning or even earlier.

Several (*n* = 10) studies specifically evaluated the effects of an early exposure to EE on synaptic plasticity. De Bartolo et al. [62] reported that a 100-day-long exposure to multidimensional EE—starting from weaning—induced a significant increase in dendritic spine density and size of Purkinje cells both in the vermis and in the hemispheres of adult rats. Such indices of synaptogenesis indicate a substantial strengthening of cerebellar circuitries. Moreover, Kim and colleagues [63] revealed that a 28-day-long exposure to multidimensional EE—starting at 3 weeks of age—induced a selective increase in parallel fiber-to-Purkinje cell synapses of same dendritic origin in mice cerebellum, indicating local synaptic strengthening aimed at the refinement of preexisting cerebellar networks. Conversely, previous studies did not report cerebellar synaptogenesis after early exposure to multidimensional EE. In mice, after a 30-day-long exposure to multidimensional EE from 28 days onwards, Nithianantharajah et al. [64] found unchanged cerebellar levels of synaptophysin, an integral membrane protein in synaptic vesicles. In agreement with this Pascual and Bustamante [65] failed to find changes in rat vermal Purkinje cell dendritic outgrowth after a 10-day-long exposure to multidimensional EE (starting from weaning). The finding of unchanged anxiety-like behavior was associated with such a neural correlate.

The early exposure to EE has also been demonstrated to induce plastic rearrangement in cerebellar molecular factors, such as the neurotrophins brain-derived neurotrophic factor (BDNF) and nerve growth factor (NGF), factors known to be strongly involved in neuronal survival and activity-dependent plasticity. In particular, Angelucci et al. [57] found increased BDNF and NGF expression in the cerebellum of rats exposed to multidimensional EE from weaning for about 120 days. In a more complex investigation, Vazquez-Sanroman et al. [66] analyzed cerebellar BDNF expression in mice exposed to multidimensional EE (but without running wheels, a key element for physical activity enhancement) starting from weaning and lasting for varying periods (1, 4, and 8 weeks). The analysis was performed through immunostaining and immunoblotting, which analyzed the expression of both immature and mature BDNF proteins. After 1 week of exposure, BDNF immunoreactivity was found to be increased only at the granular layer. After 4 and 8 weeks, BDNF immunoreactivity increased at both granular and Purkinje layers. As for the two BDNF protein isoforms, they were both unchanged after 1 week of EE exposure, whereas they were both increased after 8 weeks of EE exposure.

As for neurotransmitter expression, an investigation was conducted by Naka et al. [67] in mice exposed to multidimensional EE for 40 days starting from 28 days of age. They found increased noradrenaline but unchanged serotonin expressions in the cerebellum.

In addition, cerebellar chromatin levels, involved in RNA synthesis, also appear to be influenced by an early exposure to multidimensional EE. Uphouse [68] reported that in rats exposed to multidimensional EE (without running wheels) for 32 days from 28 days of age, cerebellar chromatin levels were increased. However, Uphouse and Tedeschi [69] reported that such change was not present after 60 days of the same treatment.

Finally, Eshra et al. [70] investigated the effects of the exposure of mice to multidimensional EE from birth to 70th–80th postnatal days on cerebellar electrophysiology. Authors showed a higher granule cell firing frequency induced by EE. This electrophysiological alteration was accompanied by enhanced motor performance.

A few (*n* = 2) studies investigated the effects on cerebellar structure and function of the exposure of animals to EE occurring *later* in life. Scholz et al. [71] analyzed the effects of exposing adult mice (7 weeks old) to 24 h or 21 days of multidimensional EE mainly based on a three-level maze, frequently rearranged. The authors reported a decrease in cerebellar volume, as revealed by in vivo and ex vivo MRI. The volume loss was interpreted to be associated with the synaptic pruning aimed at refining cerebellar circuitry functioning. After the 21-day-long exposure, such a volume change was associated with improved spatial learning. Furthermore, Horvat et al. [72] investigated the effects of a 21-day-long exposure to multidimensional EE (without running wheels) of 6-month-old rats. The authors reported increased expression of pituitary adenylate cyclase activating polypeptide, which regulates multifarious physiological and pathophysiological processes and exerts neuroprotective action.

Details on the studies cited in this section are provided in Table 1.

## 4. Neuroprotective Effects of Environmental Enrichment on the Cerebellum

A few (*n* = 5) studies investigated in animal models the neuroprotective effects of the exposure to multidimensional EE before damage occurs with the specific aim of analyzing what happens in the cerebellum. Ultimately, evidence is available on EE neuroprotective effects in models of Rett syndrome and in a model of cerebellar trauma.

*Rett syndrome* is a disease predominant in females mainly provoked by mutations in the X-linked gene for methyl CpG-binding protein 2 (Mecp2). This syndrome is characterized by an postnatal development typical in the early phases followed by the progressive loss of the acquired motor and cognitive skills, a deficit usually appearing between 6 and 18 months of age [73]. The Mecp2 mutant mice show many of the deficits observed in patients affected by Rett syndrome, such as motor impairments and alterations in social, cognitive, and emotional behavior [74]. Kondo et al. [75] investigated the effects of early exposure to multidimensional EE (without manipulation of the social factor) on hemizygous male and heterozygous female Mecp2^tm1Tam^ mice. Males (in which the symptoms show more rapid onset and progression) were exposed to EE starting from 28 days of age and lasting for 6 weeks. Females were exposed to EE from 28 days of age for 26 weeks. In hemizygous males, after the exposure to EE, unchanged BDNF expression was found in the cerebellum, in association with unchanged locomotor activity and motor coordination. In heterozygous females, although middle-term evaluations revealed that EE prevented early onset of motor coordination deficits, when the evaluation was carried out at the end of the exposure (after 26 weeks) EE failed in reversing motor coordination deficits, and cerebellar BDNF expression was found not significantly different in respect to controls reared in standard conditions. Locomotor activity was already unaffected by EE after 11 weeks of exposure. In male Mecp2^1lox^ mice, Nag et al. [76] showed that early exposure to multidimensional EE (from 21st postnatal day for 23 days—without manipulation of the social factor) did not affect cerebellar volume, motor coordination, and contextual or cued fear conditioning. However, locomotor deficits were prevented. Finally, Lonetti et al. [77] investigated the effects of a 50-day-long exposure to multidimensional EE on male Mecp2^tm1Jae^ mice, starting from 10 days of age. In this case, the authors reported that density of inhibitory synapses was higher in mutant mice than in wild-type controls and was further increased in mutant animals exposed to EE. This finding was accompanied by the prevention of deficits in motor coordination and motor learning.

A couple of studies investigated the effects of the exposure to multidimensional EE before the occurrence of a *hemicerebellectomy*, a model in which the animals are surgically deprived of a half of the vermis and one entire hemisphere. Lesioned animals show characteristic postural and locomotor asymmetries of cerebellar origin. Complex motor behavior, spatial learning, and memory performance are impaired. Typically, postural and locomotor symptoms are almost completely compensated after about 3 weeks. However, complex motor behavior and spatial performance remain defective [78]. The almost complete compensation of postural and locomotor deficits shown by lesioned animals is accompanied by plastic rearrangements in the spared hemivermis and hemisphere. Namely, Purkinje cell spine size augments in both regions. Moreover, spine density appears rearranged (decreased in the hemivermis and increased in the hemisphere) in order to maintain an homeostatic equilibrium in synaptic transmission by responding to the functional rewiring of the connectivity of the two cerebellar regions [4]. As for neurotrophin expression, both NGF and BDNF levels appear increased in the spared hemicerebellum of lesioned animals [79]. Gelfo et al. [4] showed that when animals were previously exposed to multidimensional EE (from 21st postnatal day for about 4 months), the compensation of locomotor and postural deficits was anticipated by at least one week. In addition, motor behavior, spatial learning, and memory performance were completely restored. As for cerebellar circuitry, previously enriched animals maintained the increase in Purkinje cell dendritic spine size and density induced by the EE, without showing the further increase elicited by the lesion. At the molecular level, Gelfo et al. [79] demonstrated that in the same model, NGF levels were further increased in the spared hemicerebellum of previously enriched animals. In contrast, BDNF levels were not further increased compared to the ones showed by non-enriched lesioned animals.

Details on the studies cited in this section are provided in Table 2.

## 5. Therapeutic Effects of Environmental Enrichment on the Cerebellum

A slightly larger number of studies (*n* = 7) investigated EE therapeutic effects in animal models after damage with the specific aim of analyzing what happens in the cerebellum.

Therapeutic effects of the exposure to multidimensional EE have been investigated in several models of prenatal exposure to detrimental factors, such as alcohol, stress, and betamethasone. *Prenatal exposure to alcohol* (carried out by exposing the mother to it during gestation) induces significant alterations in brain areas, particularly in the cerebellum, accompanied by abnormalities in behavior and cognition [80]. Parks et al. [81] prenatally exposed male and female rats to alcohol and then to multidimensional EE (without running wheels) starting from weaning for a period of 42 days. The authors reported that NGF and neurotrophin-3 (NT-3) vermal expression were altered by prenatal exposure to alcohol. The exposure to EE did not affect NGF levels but increased NT-3 expression at cerebellar level. *Stressful prenatal experiences* (caused by exposing the mother to them during gestation) are reported to induce neuropsychiatric disorders and cerebellar alterations, particularly affecting the morphology of Purkinje cells [82,83]. Pascual et al. [84] prenatally exposed male mice to restraint stress and then to multidimensional EE starting from weaning for 30 days. EE restored vermal Purkinje cell dendritic arborizations, which did not show stress-induced deterioration. In association, authors reported that enriched animals exhibited reduced anxiety-like behavior in comparison to standard reared controls. Finally, *prenatal exposure to betamethasone*, a corticosteroid commonly used in obstetrics (carried out by exposing the mother to it during gestation), constitutes a risk factor for the development of behavioral, cognitive, and neurological alterations [85]. Valencia et al. [86] reported that prenatal treatment with betamethasone induced alterations of vermal synaptophysin levels in young adult rats. The exposure to multidimensional EE starting from weaning for 18 days was able to restore standard vermal synaptophysin levels and recover motor coordination.

Two studies performed in the last century analyzed the effects of exposure to an enriched environment in animals previously undernourished in the first postnatal period. *Malnutrition in early life* is reported to provoke neural changes in both humans and animals by impairing brain morphology and functionality. A selective decrement of cerebellar structure is described [87] in association with alterations in cognition and behavior [88]. McConnel et al. [87] exposed male and female rats to undernutrition from birth to 30th postnatal day and then to a 140-day-long period of multidimensional EE (without running wheels). After EE, the authors described a restoration of cerebellar weight in females but not in males. In contrast, Lima et al. [88] undernourished male rats from birth to 50th postnatal day and exposed these animals to handling from birth for 21 days and then to multidimensional EE (without running wheels) for 27 days. After the exposure to EE, the enriched animals showed increased cerebellar weight in comparison to the non-enriched controls, with increased total deoxyribonucleic acid (DNA) amount and unchanged total ribonucleic acid (RNA) amount. Notably, enriched animals showed a reduction in aversiveness in the inhibitory avoidance test.

*Aging* is known to provoke cognitive decline and alterations in brain structure and function, including changes in cerebellar levels of nitric oxide, which is produced by neurons and acts as a neurotransmitter to regulate functions ranging from digestion and blood flow to memory and vision [89]. Tomiga et al. [90] investigated in a model of aging (male mice of 19.5 months of age) the effects of the exposure to 6 weeks of multidimensional EE. The authors reported that nitric oxide synthase expression increased in the cerebellum of aged mice but was reduced by the exposure to EE. In parallel, reduced anxiety-like behaviors were reported in enriched aged mice.

Finally, the therapeutic effects of EE have also been investigated in a mouse model of *hereditary cerebellar degeneration*, the Lurcher mutant mice. In this model, Purkinje cells almost completely degenerate within 3 months of age. In addition, massive alterations of the other cerebellar cell populations occur. At a functional level, Lurcher mutant mice show cerebellar ataxia with cognitive and behavioral deficits [91]. In a recent study, Salomova et al. [92] exposed 8-week-old male and female Lurcher mutant mice to 9 weeks of multidimensional EE. At the end of the treatment, although some reduction of behavioral disinhibition was found in enriched animals, motor performance remained impaired and cerebellar BDNF remained unchanged.

Details on the studies cited in this section are provided in Table 3.

## 6. Conclusions

**Figure 2:**
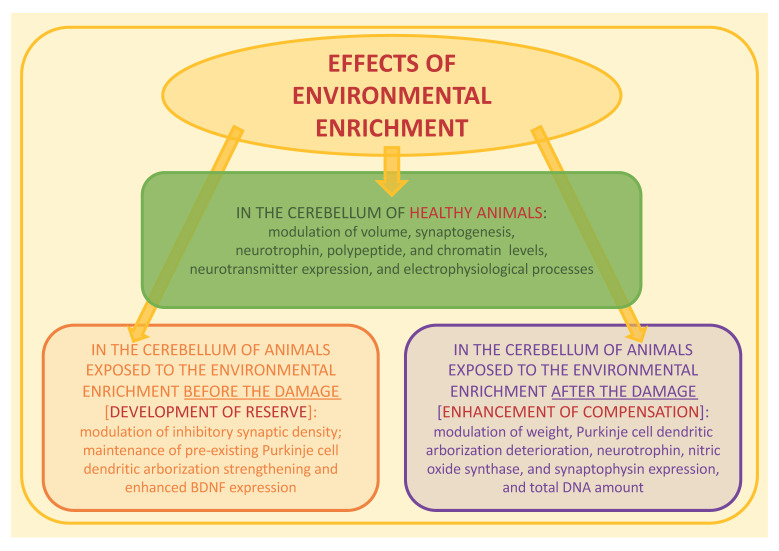
The panel synthesizes the evidence provided by animal models on the effects of environmental enrichment exposure on the cerebellum, taking into account the studies on healthy subjects (box in the middle) and on animals exposed to EE before (box at the bottom on the left side) and after (box at the bottom on the right side) a damage involving cerebellar functionality. BDNF: brain-derived neurotrophic factor; DNA: deoxyribonucleic acid.

The aim of this review was the collection and the synthesis of the evidence provided by animal models on the effects of EE exposure on cerebellar structure and function, taking into account the studies on healthy subjects and on animals exposed to EE before and after damage involving cerebellar functionality. Figure 2 shows a panel summarizing the evidence synthesized in this review. To the best of our knowledge, this is the first review of the literature on the effects of exposure to EE specifically devoted to such a topic.

On the whole, the evidence collected suggests that EE is able to enhance cerebellar compensation and develop cerebellar reserve. However, the limited indications available do not still offer a clear and coherent framework.

As for the healthy animals, most findings concern the effects of *early* exposures to EE, starting from the birth or the weaning of the animals. Several studies report that EE induces plastic rearrangements in the cerebellar regions, describing changes in synaptogenesis [62,63], neurotrophin levels [57,66], neurotransmitter expression [67], and chromatin levels [68], as well as electrophysiological modifications [70]. However, opposing evidence reports the absence of EE effects on synaptogenesis [64,65], neurotrophin [66] and neurotransmitter expression [67], and chromatin levels [69]. As regards the exposure of animals to EE *later* in life, within the limited indications available, the changes in cerebellar volume and polypeptides associated with improved spatial learning described in mice and rats exposed to EE in adulthood should be mentioned [71,72]. On the whole, it is possible to include cerebellar rearrangement among the beneficial effects of EE, even if more systematic research and more consistent results are needed to understand the mechanisms involved. Stamenkovic et al. [93] advanced that the extracellular matrix glycoprotein tenascin-C contributes to the regulation of cerebellar structural plasticity, also in response to EE, and that the interaction between such a glycoprotein and the degrading enzyme matrix metalloproteinase-9 may be critical for the occurrence of EE-driven rearrangement. Interestingly, some evidence has also been provided on transgenerational effects of the exposure to EE on cerebellar plasticity. Pre-reproductive maternal exposure to EE is reported to result in enhanced cerebellar BDNF and NGF expression in pups both at birth and at weaning, associated with earlier acquisition of complex motor abilities [94].

As for the evidence available on the beneficial neuroprotective action of EE in developing a cerebellar reserve that strengthens the capacity of the individual to cope with brain damage, studies have been conducted with reference to two pathological conditions involving cerebellar functionality, namely Rett syndrome (modeled in transgenic mice) and cerebellar trauma (modeled by unilateral cerebellar ablation). In mice transgenic models of Rett syndrome, it has been demonstrated that the early exposure to EE was able to at least partially prevent motor deficits [75,76,77] and to modulate cerebellar inhibitory synaptic density [77]. However, unchanged cerebellar volume [76] and BDNF expression [75] were also reported. On the other hand, in rats exposed to EE from weaning before hemicerebellectomy, it has been demonstrated that in association with an accelerated motor recovery, Purkinje cells maintained the strengthened rearrangement induced by the EE, without showing most changes present in standard reared lesioned animals [4]. Similarly, the augmented BDNF expression induced by the EE was not further rearranged as a consequence of the lesion [79]. This pattern seems to indicate that further morphological rearrangements aiming to support cerebellar compensation may not be needed in a brain previously empowered by the exposure to EE. It has been advanced that this pattern was accompanied by an EE-driven massive rearrangement of the rest of the brain, involving neocortical and striatal neural morphology [31,60] and neocortical neurotrophin expression [79].

Finally, evidence has also been provided about the therapeutic action of EE in enhancing spontaneous cerebellar compensation following damage, as well as when the exposure occurs and the damage is already present. EE therapeutic effects have been investigated in the presence of a number of different pathological patterns. The exposure to EE in periods following weaning affects cerebellar structure and function after prenatal exposure to detrimental factors, such as alcohol [81], stress [84], and betamethasone [86], by inducing changes at molecular and supramolecular levels in association with reduction of behavioral symptoms. Beneficial EE effects are also documented after undernourishment in early life, in which is reported the reduction of aversiveness in the inhibitory avoidance and change in cerebellar weight and DNA total amount [87,88]. Beneficial effects are also found in a mouse aging model, in which EE reduces anxiety-like behaviors and nitric oxide synthase expression [90]. Finally, in a mouse ataxia model, EE was found to reduce behavioral disinhibition but failed to change motor performance and cerebellar BDNF expression [92]. Additionally, in the case of exposure to EE after the damage, it has been advanced that the enhancement of cerebellar compensation that follows EE may be due to the synergistic action of other brain areas, such as the striatum [95].

In conclusion, evidence provided by animal studies supports the beneficial action of enhanced stimulations in developing a cerebellar reserve that is able to potentiate spontaneous cerebellar capacities to compensate for deficits induced by brain injury or neurodegeneration. Such a beneficial action is also present when the complex stimulations are applied after the occurrence of the damage. However, studies addressing this issue with a specific interest in the cerebellum are still scarce, and the poor evidence provided means that large areas of inconsistency and lack of clarity remain. Moreover, the studies were performed at various points across a long period of time, and thus a significant number did not use modern innovative techniques and methodological standards. Thus, it would be advisable that increased attention is devoted to this issue, since it could be very relevant for management of cerebellar patients.

Indeed, in terms of clinical implications, deepening the biological bases of experiential stimulation effects on the development of a cerebellar reserve, to be spent in case of damage, and on the enhancement of cerebellar spontaneous compensation after damage may be crucial for two key reasons. Firstly, such a line of research could shed some light on mechanisms involved in cerebellar reserve and compensation of human pathologies that involve cerebellar functionality [96]. Secondly, understanding the direction and extent of the cerebellar rearrangements, as well as the timing of effective interventions, may provide a good basis for tuned and effective clinical interventions in humans. Human studies described the high cerebellar capacity to compensate for damage due to acute focal lesions or diffuse neurodegeneration. Such a compensation occurs in the so-called “restorable stage”, in which intact cerebellar and extra-cerebellar areas are still able to cope with damage [55]. This could be an ideal period in which plastic factors may act to improve cerebellar compensation capacity. However, human studies devoted to analyzing the effects of experiences occurring before or after an injury on cerebellar capacity to compensate for a damage are still scarce. Some indications are provided by studies on the beneficial effects of physical activity on motor and cognitive performances of older adults (65–86 years) by documenting that such effects are accompanied by a more targeted and less widespread cerebellar activation [97]. Moreover, some evidence has been provided in patients with cerebellar degeneration, in whom physical training improves motor deficits. Such an effect would be mediated by increased cerebellar volumes accompanied by increased gray matter volumes of non-affected neocortical regions [98]. Further, if applied when residual cerebellar reserve is present, even non-invasive cerebellar stimulation techniques are able to potentiate compensation in many human cerebellar pathologies [99]. However, on the whole, as also reported by human secondary studies, the neuronal mechanisms underlying experiential and non-invasive stimulations of the development of cerebellar reserve and of the enhancement of compensation are still largely unknown [100]. Further animal and human primary and systematic secondary studies may provide valid suggestions on mechanisms involved in such phenomena.

## Figures and Tables

**Table 1:** Studies on environmental enrichment’s effects in healthy animals.

Reference	Species *(Age or Weight at the Start of* *Environmental Enrichment)*	Environmental Enrichment Type *(Duration)*	Environmental Enrichment Effects on Cerebellar Structure and Function *(Age at the Effect Evaluations)*
Uphouse, 1978 [68]	Male Fischer rats (28 *days*)	Environmental enrichment —without running wheels; with novelty manipulation (32 *days*)	Increased chromatin level (2 *months*)
Uphouse and Tedeschi, 1979 [69]	Male Fischer rats (28 *days*)	Environmental enrichment —without running wheels; with novelty manipulation (60 *days*)	Unchanged chromatin level (*about* 3 *months*)
Naka et al., 2002 [67]	Male ICR mice (28 *days*)	Environmental enrichment —with running wheels and novelty manipulation (40 *days*)	Increased noradrenaline expression; unchanged serotonin and metabolites expression *(about* 2 *months*)
Nithianantharajah et al., 2004 [64]	Female C57BL/6 mice (28 *days*)	Environmental enrichment —with running wheels and novelty manipulation (30 *days*)	Unchanged synaptophysin level (*about* 2 *months*)
Angelucci et al., 2009 [57]	Male Wistar rats (21 *days*)	Environmental enrichment —with running wheels and novelty manipulation *(about* 120 *days*)	Increased nerve growth factor (NGF) and brain-derived neurotrophic factor (BDNF) levels (140 *days*)
Pascual and Bustamante, 2013 [65]	Male Sprague–Dawley rats (22 *days*)	Environmental enrichment —with running wheels and novelty manipulation (10 *days*)	Unchanged anxiety-like behavior (*33–34 days*); unchanged vermal Purkinje cell dendritic outgrowth (36 *days*)
Vazquez-Sanroman et al., 2013 [66]	Male Balb/c AnNHsd mice (21 *days*)	Environmental enrichment —without running wheels; with novelty manipulation (1/4/8 *weeks*)	After 1 week: unchanged pro-BDNF and mature BDNF proteins; increased BDNF immunoreactivity at granular layer; unchanged BDNF immunoreactivity at Purkinje layer (4 *weeks*) After 4 weeks: increased BDNF immunoreactivity at granular and Purkinje layers (7 *weeks*) After 8 weeks: increased pro-BDNF and mature BDNF proteins; increased BDNF immunoreactivity at granular and Purkinje layers (11 *weeks*)
De Bartolo et al., 2015 [62]	Male Wistar rats (21 *days*)	Environmental enrichment —with running wheels and novelty manipulation (*about* 100 *days*)	Increased cerebellar Purkinje cell dendritic spine density and size (*about* 120 *days*)
Horvat et al., 2015 [72]	Male Wistar rats (6 *months*)	Environmental enrichment —without running wheels; with novelty manipulation (21 *days*)	Increased pituitary adenylate cyclase activating polypeptide (PACAP) 27 expression; unchanged PACAP 38 expression (27 *weeks*)
Scholz et al., 2015 [71]	Male C57BL/B6 mice (7 *weeks*)	Environmental enrichment —with running wheels and novelty manipulation—based on a three-level maze, without objects (24 *h;* 21 *days*)	Improved spatial learning (10 *weeks*); decreased volume (about 7 *weeks;* 10 *weeks*)
Eshra et al., 2019 [70]	C57BL/6 mice *(at birth)*	Environmental enrichment —with running wheels and novelty manipulation (70–80 *days*)	Improved motor performance; higher granule cell firing frequency (70–80 *days*)
Kim et al., 2019 [63]	C57BL/6 mice (3 *weeks*)	Environmental enrichment —with running wheels and novelty manipulation (28 *days*)	Selective increase in parallel fiber-to-Purkinje cell synapses of same dendritic origin, with local synaptic strengthening (7 *weeks*)

Note: unless otherwise specified, the described effects involve the entire cerebellar structure.

**Table 2:** Studies on neuroprotective environmental enrichment effects in pathological animal models.

Reference	Species and Pathological Model *(Age or Weight at the Start of* *Environmental Enrichment)*	Environmental Enrichment Type *(Duration)*	Environmental Enrichment Effects on Cerebellar Structure and Function *(Age at the Effect Evaluation)*
Kondo et al., 2008 [75]	Hemizygous male and heterozygous female Mecp2^tm1Tam^ mice; model of Rett syndrome (more rapid onset and progression of symptoms in males) (28 *days*)	Environmental enrichment —with running wheels and novelty manipulation; without social manipulation (*Males:* 6 *weeks;* *Females:* 26 *weeks*)	In males: unchanged locomotor activity (6; 9 *weeks*) and motor coordination (7; 8; 9 *weeks*); unchanged BDNF expression (10 *weeks*)
			In females: prevention of early motor coordination deficits (20; 23; 26 *weeks*); unchanged locomotor activity (15 *weeks*) and late motor coordination (29 *weeks*); unchanged BDNF expression (30 *weeks*)
Nag et al., 2009 [76]	Male Mecp2^1lox^ mice; model of Rett syndrome (21 *days*)	Environmental enrichment —with running wheels and novelty manipulation; without social manipulation (23 *days*)	Prevention of locomotor deficits; unchanged motor coordination and contextual or cued fear conditioning (29–43 *days*); unchanged volume (44 *days*)
Lonetti et al., 2010 [77]	Male Mecp2^tm1Jae^ mice; model of Rett syndrome (10 *days*)	Environmental enrichment —with running wheels and novelty manipulation (50 *days*)	In males: prevention of motor coordination and motor learning deficits (30–60 *days*); increased inhibitory synaptic density (52 *days*)
Gelfo et al., 2011 [79]	Male Wistar rats; model of cerebellar trauma (hemicerebellectomy at 75th postnatal day) (21 *days*)	Environmental enrichment —with running wheels and novelty manipulation *(about* 4 *months*)	Accelerated motor recovery (1–42 *post-operative days*); increased NGF expression; unchanged BDNF expression (5 *months*)
Gelfo et al., 2016 [4]	Male Wistar rats; model of cerebellar trauma (hemicerebellectomy at 75th postnatal day) (21 *days*)	Environmental enrichment —with running wheels and novelty manipulation (*about* 4 *months*)	Accelerated motor recovery and restoration of complex motor behaviors (1–56 *post-operative days*); increased spatial learning and memory performance (*about* 4 *months*); maintaining of Purkinje cell dendritic spine density and size (*about* 4.5 *months*)

Note: unless otherwise specified, the described effects involve the entire cerebellar structure.

**Table 3:** Studies on the therapeutic environmental enrichment effects in pathological animal models.

**Reference**	**Species and Pathological Model** ***(Age or Weight at the Start of*** ***Environmental Enrichment)***	**Environmental Enrichment Type** ***(Duration)***	**Environmental Enrichment Effects on Cerebellar Structure and Function** ***(Age at the Effect Evaluation)***
McConnell et al., 1981 [87]	Male and female rats; undernourished from birth to 30th postnatal day (30 *days*)	Environmental enrichment —without running wheels; with novelty manipulation (140 *days*)	In males: no restoration of cerebellar weight (170 *days*) In females: restoration of cerebellar weight (170 *days*)
Lima et al., 1998 [88]	Male Wistar rats; undernourished from birth to 50th postnatal day *(handling from birth to* 21 *days; then environmental enrichment*)	Environmental enrichment —without running wheels and novelty manipulation (27 *days*)	Reduction of aversiveness in the inhibitory avoidance test (47 *days*); Increased weight; increased total deoxyribonucleic acid (DNA) amount; unchanged total ribonucleic (RNA) amount (50 *days*)
Parks et al., 2008 [81]	Male/female Sprague–Dawley rats; prenatal alcohol exposure from gestational day 8 to gestational day 20 (21 *days*)	Environmental enrichment —without running wheels; with novelty manipulation (42 *days*)	Unchanged vermal NGF expression; increased vermal (neurotrophin-3) NT-3 expression (66 *days*)
Pascual et al., 2015 [84]	Male CF-1 mice; exposed to prenatal restraint stress from gestational day 14 to gestational day 21 (22 *days*)	Environmental enrichment —with running wheels and novelty manipulation *(30 days)*	Reduced anxiety-like behavior; rescuing of the vermal Purkinje cell dendritic deterioration (*82 days*)
Tomiga et al., 2016 [90]	Male C57BL/6J mice; model of aging (19.5 *months*)	Environmental enrichment —with running wheels and novelty manipulation (6 *weeks*)	Reduced anxiety-like behavior; reduced nitric oxide synthase expression (21 *months*)
Valencia et al., 2019 [86]	Male Sprague–Dawley rats; exposed to prenatal treatment with betamethasone on gestational day 20 (21 *days*)	Environmental enrichment —with running wheels and novelty manipulation (18 *days*)	Restored motor coordination; restored vermal synaptophysin level (52 *days*)
Salomova et al., 2021 [92]	Male and female Lurcher mutant mice (8 *weeks*)	Environmental enrichment —with running wheels and novelty manipulation (9 *weeks*)	Unchanged motor performance; reduction in behavioral disinhibition (15–16 *weeks*); unchanged BDNF expression (17 *weeks*)

Note: unless otherwise specified, the described effects involve the entire cerebellar structure.

## Data Availability

The concept reported in this manuscript is not associated with raw data.
